# The Impact of Excessive Gestational Weight Gain on Adverse Perinatal Outcomes: A Systematic Review

**DOI:** 10.3390/jcm14041197

**Published:** 2025-02-12

**Authors:** Eleni Kalli, Anastasios Potiris, Ekaterini Domali, Athanasios Zikopoulos, Nikolaos Kathopoulis, Eirini Drakaki, Nikolaos Machairiotis, Konstantinos Louis, Athanasios Gkirgkinoudis, Chrysi Christodoulaki, Athanasios Zachariou, Charikleia Skentou, Angeliki Gerede, Konstantinos Zikopoulos, Peter Drakakis, Periklis Panagopoulos, Sofoklis Stavros

**Affiliations:** 1 Medical School, National and Kapodistrian University of Athens, 115 27 Athens, Greece; eleni.g.kalli@gmail.com; 2 Third Department of Obstetrics and Gynecology, University General Hospital “ATTIKON”, Medical School, National and Kapodistrian University of Athens, 124 62 Athens, Greece; thanzik92@gmail.com (A.Z.); nikolaosmachairiotis@gmail.com (N.M.); kostaslouisss@gmail.com (K.L.); pdrakakis@med.uoa.gr (P.D.); perpanag@med.uoa.gr (P.P.); sfstavrou@med.uoa.gr (S.S.); 3 First Department of Obstetrics and Gynecology, Alexandra Hospital, Medical School, National and Kapodistrian University of Athens, 115 28 Athens, Greece; kdomali@yahoo.fr (E.D.); nickatho@gmail.com (N.K.); eirinidrak@med.uoa.gr (E.D.); tgkirgki@gmail.com (A.G.); 4 Department of Obstetrics and Gynecology, Chania General Hospital “St. George”, 733 00 Chania, Greece; christodoulakichr@hotmail.com; 5 Department of Urology, School of Medicine, Ioannina University, 451 10 Ioannina, Greece; zahariou@otenet.gr; 6 Department of Obstetrics and Gynecology, Medical School, University of Ioannina, 451 10 Ioannina, Greece; haraskentou@uoi.gr (C.S.); kzikop22@gmail.com (K.Z.); 7 Department of Obstetrics and Gynecology, Democritus University of Thrace, 691 00 Campus, Greece; agerede@otenet.gr

**Keywords:** weight gain, pregnancy, adverse perinatal outcomes, neonate

## Abstract

**Background/Objectives:** The purpose of this study was to systematically review the potential effects of a pregnant woman’s excessive gestational weight gain on adverse perinatal outcomes affecting the mother and the fetus/neonate. **Methods:** Medline/PubMed, Scopus, CADTH Grey Matters and National Archive of PhD Theses were systematically searched for all relevant studies published. Assessments of the risk of bias in the included studies were made according to the tool “The Newcastle–Ottawa Scale (NOS)”. **Results:** Five publications met all the inclusion criteria and were included in this review. The risk of bias in all the included studies was low. One study supports the detrimental effect of excessive gestational weight gain on the risk of gestational diabetes mellitus, one study on the risk of preterm birth, five studies on the risk of macrosomia-high birthweight of the neonate, three studies on the risk of a large-for-gestational-age neonate, three studies on the risk of hypertensive disorders of pregnancy, one study on the risk of gestational hypertension and preeclampsia, five studies on the risk of delivery by cesarean section and one study on the risk of neonatal hypoglycemia requiring treatment. One study supports the beneficial effect of excessive gestational weight gain on the risk of preterm birth, two studies on the risk of a small-for-gestational-age neonate and two studies on the risk of low birthweight of the neonate. **Conclusions:** The study presents the most recent and strong evidence regarding the negative effect of excessive gestational weight gain on most adverse perinatal outcomes. However, excessive gestational weight gain has a beneficial effect in a very limited number of outcomes.

## 1. Introduction

Excessive gestational weight gain (EGWG) is a major public health issue, specifically in certain areas of the world [1]. The prevalence of excessive gestational weight gain worldwide is 39.4%, with the highest prevalence documented in North America (50.6%) and the lowest in Asia (20.2%). The aforementioned prevalence differs also depending on the pre-pregnancy body mass index status of the pregnant woman [2]. Excessive gestational weight gain concerns 21% of underweight women, 36% of normal-weight women, 64% of overweight women and 60% of obese women before pregnancy.

In many studies, EGWG is defined as gestational weight gain exceeding the 2009 Institute of Medicine gestational weight gain guidelines threshold [3]. This threshold varies based on the pre-pregnancy body mass index status of the woman. For instance, for an underweight woman with a singleton pregnancy, this could be gaining more than 18 kg, whereas for an obese woman, the equivalent is gaining more than 9 kg. Among the detrimental and protective factors for EGWG, a woman’s weight at the time of conception is the most important predictor, whereas women who are overweight or obese are at higher risk for it [4]. According to that review, race and educational level are also important factors for EGWG. Black and Hispanic women exhibit lower rates of excessive gestational weight gain compared to white women. However, obesity rates are higher in the former. Higher educational level was found to be protective for white women, but for Hispanic women and those with lower socioeconomic status, it was found to be detrimental.

Adverse perinatal outcomes refer to the perinatal period, which begins at the 22nd week of gestation and ends 7 days after delivery according to the World Health Organization. These complications affect the pregnant woman before and after delivery, as well as the neonate. Some of the most common perinatal complications are gestational diabetes mellitus which complicates almost 14% of all pregnancies worldwide [5], preterm birth (<37 weeks of gestation) which refers to 4–16% of neonates born in 2020, gestational hypertensive disorders (gestational hypertension, preeclampsia, eclampsia) which refer to 4.1% to 19.4% of all pregnancies worldwide [6] and delivery via cesarean section [7]. Furthermore, antenatal, perinatal and postpartum depression complicates 26.3–28.5% of all pregnancies [8]. Neonatal complications such as macrosomia (7.8% of live births in the United States) [9], small size for gestational age (incidence of 10–25% worldwide) [10] and neonatal hypoglycemia (up to 15% of newborns) [11] are also equally common.

A significant number of previous and recent systematic reviews and meta-analyses [12,13,14,15,16,17] have investigated the correlation between excessive gestational weight gain and perinatal complications in various subgroups of the population (study sample from 24 low- and middle-income countries, sub-Saharan Africa, Chinese women with gestational diabetes mellitus, twin pregnancies, women with gestational diabetes mellitus from seven different countries, healthy women with a singleton pregnancy and a body mass index of 18.5 kg/m^2^ or more from 16 countries). Some of them have concluded a higher risk of perinatal complications such as preterm birth [17], a large-for-gestational-age neonate [12,14,16,17], macrosomia [12,17], cesarean section [12,16], gestational hypertension [12], preeclampsia [13] and lower risk of a small-for-gestational-age neonate [12,16,17] with EGWG. Nevertheless, others have concluded statistically insignificant results for the same outcomes and specifically for preterm birth [12,13,14,16], macrosomia [15], cesarean section [13,14], a small-for-gestational-age neonate [13,14] and preeclampsia [14]. Because of these inconclusive results, we deemed that this systematic review would be a valuable contribution to the research.

Hence, the aim of the present systematic review is to investigate and enlighten the potential correlation between excessive gestational weight gain and adverse perinatal outcomes. Ultimately, the aim of our study is to assess if excessive gestational weight gain during pregnancy affects the risk of adverse perinatal outcomes both for the pregnant woman/mother and the fetus/neonate.

## 2. Materials and Methods

This review was performed in accordance with the Preferred Reporting Items for Systematic Reviews and Meta-Analyses (PRISMA 2020) guidelines (available at https://www.prisma-statement.org/prisma-2020, accessed on 9 February 2025) and was registered in OSF REGISTRIES on 27 November 2024 (Registration DOI: https://doi.org/10.17605/OSF.IO/XGWJ6).

### 2.1. Inclusion and Exclusion Criteria of the Study

The initial inclusion criteria included original studies published within the last 5 years, i.e., from the year 2019 (1 January 2019) to the year 2024 (4 September 2024) and published in the English language. The PICO (Patient, Intervention, Control (comparator) and Outcome) process was implemented as guidance through the inclusion criteria:

Patient: All pregnant women of any age and state of health.

Intervention: Excessive maternal weight gain during pregnancy, i.e., maternal weight gain above the recommended limits based on body mass index category the pregnant woman belongs to (underweight, normal weight, overweight, obese). This excessive weight gain could happen and be evaluated (a) from the beginning of pregnancy until delivery, (b) from the beginning of pregnancy until some specific time(s) or other milestone(s) of pregnancy, e.g., up to the 2nd trimester, after performing a glucose tolerance test during pregnancy, etc., or (c) from this specific time/s or other milestone/s of pregnancy until delivery.

Comparator: The comparison group is normal weight gain or weight gain within the recommendations based on the body mass index category the pregnant woman belongs to (underweight, normal weight, overweight, obese) and comparing the body mass index category she belongs to (underweight, overweight, obese) with the normal-weight category, whether this weight gain is excessive or normal weight gain.

Outcome: Any perinatal complication that affects either the pregnant woman/mother or the fetus/neonate or both.

The highest and most recent level of evidence that could be found was used to answer the clinical question of the review. Overall, the exclusion criteria included systematic reviews, reviews, meta-analyses and studies published in languages other than English.

### 2.2. Search Strategy and Databases

A systematic bibliographic search for relevant references was conducted in four databases: Medline/PubMed, Scopus, CADTH Grey Matters, National Archive of PhD Theses. The initial search was carried out until 20 July 2024 for the Medline/PubMed database, 15 August 2024 for the Scopus database and 31 August 2024 for the CADTH Grey Matters and National Archive of Doctoral Theses (NADTH) databases. A repeat and final search was undertaken οn 4 September 2024 for all four databases.

The search terms used included “excessive weight gain”, “weight gain”, “BMI change”, “body mass index change”, “adverse perinatal outcomes” and “perinatal complications”, “pregnancy outcomes”, “birth outcomes” and “neonatal outcomes” with the administration of Boolean operators (OR, AND) combined with those keywords either used as presented, separately or in combination. Filters about the year or date of publication also applied. The detailed search strategy used for each electronic database can be found in Supplementary Table S1.

### 2.3. Study Selection and Data Extraction

The records identified by the search were entered into the reference management program EndNote 21, and then duplicate citations were removed. The title and abstract of all retrieved publications were screened independently by two reviewers (E.K. and A.P.), and the full text of the eligible ones was screened subsequently. If there was a study selected only by one reviewer, the decision was made by a third reviewer (S.S.). The data collection process was carried out by only one reviewer. A data extraction form with the necessary study data (characteristics, effect measures) was used. The outcomes (adverse perinatal outcomes) for which data were collected were as follows: gestational diabetes mellitus, hypertensive disorders of pregnancy, gestational hypertension, preeclampsia, preterm birth, delivery by cesarean section, abnormal growth of the fetus/neonate (macrosomia-high birth weight of the neonate, low birth weight of the neonate, large- or small-for-gestational-age neonate), other maternal conditions related to pregnancy and childbirth (antenatal, perinatal and postpartum depressive symptoms, gestational anemia), complications during normal labor, neonatal complications (neonatal hypoglycemia requiring treatment, admission to Neonatal Intensive Care Unit, congenital anomaly of the fetus/neonate).

Data analyses adjusted for confounders (where available), e.g., the relative ratio and 95% confidence interval, were collected compared to non-adjusted data analyses. When there were multiple time points of follow-up during each study, results from all-time points were collected and presented, e.g., before and after diagnosis of gestational diabetes mellitus, at the end of the 2nd trimester and at the end of the 3rd trimester.

The additional variables for which data were collected included names of authors and year of publication, study type and design, important information for the sample (size, subgroup of the population, e.g., women with gestational diabetes mellitus), country or geographical area of the study, information about exposure variable, outcomes assessed by the study, important findings of the study. The recording of incomplete data, e.g., the number of pregnant women who had excessive weight gain during pregnancy, was omitted. Only data available from the studies were recorded without making any calculations or estimations.

### 2.4. Risk of Bias and Quality Assessment

The assessment of the included studies’ risk of bias was conducted by two reviewers (E.K. and A.P.). For this purpose, the tool “The Newcastle–Ottawa Scale (NOS)” was used (available at https://www.ohri.ca/programs/clinical_epidemiology/oxford.asp, accessed on 15 November 2024). The scale is suitable for assessing the methodological quality of cohort studies and therefore their risk of bias. It consists of three main components—assessment criteria. Briefly, the 1st is related to the selection of study groups (exposed and unexposed), the 2nd to the comparability between the 2 groups and the 3rd to the measurement/evaluation of outcomes.

## 3. Results

### 3.1. Study Selection Process

**Figure 1:**
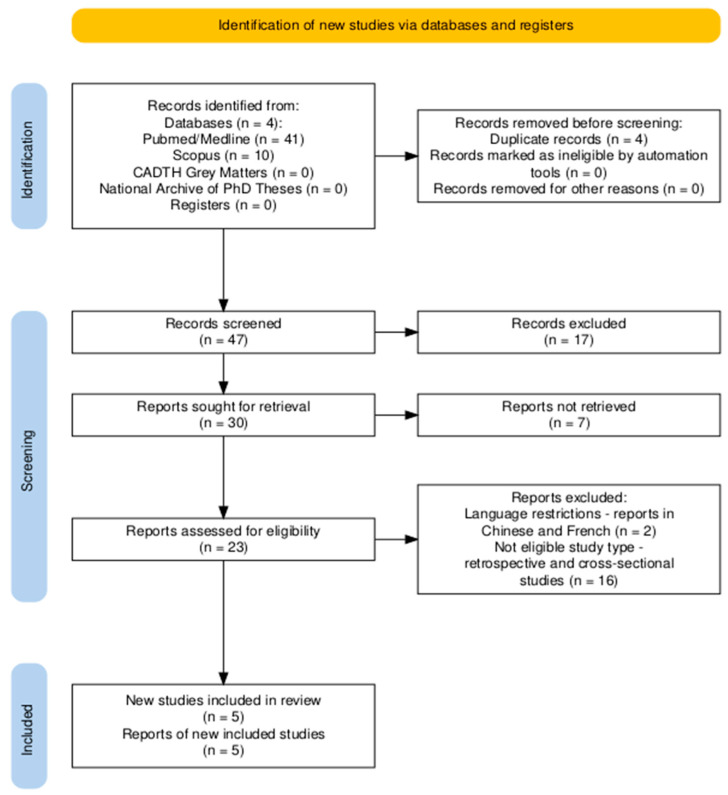
Flow diagram of the study selection process.

From the initial research, 51 articles were collected via PubMed/Medline, Scopus, CADTH Grey Matters and National Archive of PhD theses electronic databases. A total of 47 were screened by title and abstract, and 30 underwent full-text assessment. Ultimately, five articles were suitable for providing information in this literature review. The study selection process is depicted in Figure 1.

### 3.2. Characteristics of the Included Studies

Table 1 presents the main characteristics (study information, study type, geographical area of the study, sample information, perinatal outcomes assessed by each study, weight gain during pregnancy in kilograms, gestational age at delivery in weeks, mode of delivery, neonatal birth weight, Apgar score) of the five studies that were included in the review.

### 3.3. Risk of Bias Assessment

The assessment of the methodological quality of the five included studies is depicted in Table 2. All five studies were prospective cohort studies and were found to be of high quality and low risk of bias. Therefore, all five studies were included in this review.

### 3.4. Synthesis of Extracted Data

In Table 3, the direction and magnitude of excessive gestational weight gain’s effect on the risk of adverse perinatal outcome is presented by type of complication.

One study was from North America (Canada), one from South America (Brazil) and three from East Asia (Taiwan, Korea, China). The total number of women included in this review was 34,803. For nine adverse perinatal outcomes (gestational diabetes mellitus, hypertensive disorders of pregnancy, gestational hypertension, preeclampsia, delivery by cesarean section, prenatal depressive symptoms, large-for-gestational-age neonate, high-birth-weight neonate, neonatal hypoglycemia requiring treatment), an increased risk or a detrimental effect of excessive maternal weight gain during pregnancy was observed. Regarding small-for-gestational-age and low-birth-weight neonates, a reduction in the risk or a protective effect was observed. In contrast, regarding preterm birth and neonatal macrosomia, both a reduction and an increased risk were observed depending on the subgroup of the population examined (underweight and overweight/obese at the beginning of pregnancy, respectively). The highest increase in risk or detrimental effect was observed for gestational hypertension in pregnant women who were obese at the beginning of pregnancy, while the lowest increase or detrimental effect was observed in delivery by cesarean section for pregnant women who had excessive weight gain in the 2nd trimester of pregnancy. The highest risk reduction or protective effect was observed for macrosomia-high birth weight of the neonate in pregnant women who were underweight at the beginning of pregnancy, whereas the lowest reduction or protective effect was observed for preterm birth.

## 4. Discussion

In this systematic review, we investigated the correlation between a pregnant woman’s excessive gestational weight gain and its impact on different adverse perinatal outcomes. We aimed not only to determine the EGWG’s direction of effect but also to quantify EGWG’s magnitude of effect on the risk of each perinatal outcome in the general population of pregnant women as well as in its specific subgroups (underweight/overweight/obese, pregnancies with gestational diabetes mellitus).

Regarding the risk of gestational diabetes mellitus, hypertensive disorders of pregnancy, gestational hypertension, preeclampsia, delivery by cesarean section, prenatal depressive symptoms, large-for-gestational-age neonate, high-birth-weight neonate and neonatal hypoglycemia requiring treatment, an increased risk or a detrimental effect of maternal weight gain during pregnancy above the recommended limits was observed. Regarding the risk of a small-for-gestational-age neonate and low-birth-weight neonate, a decrease in risk or a protective effect of maternal weight gain during pregnancy above the recommended limits was observed. Regarding the risk of preterm birth and macrosomia-high birth weight of the neonate, the results were conflicting and differed depending on the subgroup of the population examined (reduced risk in pregnant women who were underweight at the beginning of pregnancy and increased risk in pregnant women who were overweight or obese at the beginning of pregnancy).

The risk of gestational diabetes mellitus was found to be increased with EGWG in our review, but this finding is the result of only one included study, and thus, it should be interpreted with great caution. Contrary to that finding, in the review and meta-analysis of Lipworth et al. regarding twin pregnancies, EGWG was associated with a reduced risk in overweight women [13].

In this review, the results about the risk of preterm birth were found to be conflicting and differ depending on the pre-pregnancy body mass index category of pregnant women. More specifically, we found a reduced risk of preterm birth in pregnant women who were underweight at the beginning of pregnancy and an increased risk in pregnant women who were overweight or obese at the beginning of pregnancy. Nevertheless, this result arose from the findings of only one included study, and thus, it should be interpreted with great caution. Underweight, overweight and obese women before pregnancy have been found to be at higher risk for preterm birth (<37 weeks of gestation) compared to normal-weight women [23]. The results of Perumal et al.’s meta-analysis [17] including data from 24 low- and middle-income countries are in accordance with the increased risk of preterm birth with EGWG. In contrast, four other systematic reviews–meta-analyses concluded statistically insignificant results regarding the correlation between EGWG and preterm birth: the review and meta-analysis of He et al. [14] regarding pregnant women with gestational diabetes mellitus, review and meta-analysis of Lipworth et al. [13] regarding twin pregnancies, systematic review and meta-analysis of Zhu et al. [12] including pregnant women from China with gestational diabetes mellitus and meta-analysis of Rogozinska [16] which analyzed individual participant data of healthy women with a singleton pregnancy from 36 randomized trials (16 countries).

Regarding the risk of preeclampsia, our review’s results are aligned with the results of the systematic review and meta-analysis of Lipworth et al. [13] regarding twin pregnancies which concluded that EGWG is associated with an increased risk of it for all body mass index categories. In contrast, in the review and meta-analysis of He et al. [14] including pregnant women with gestational diabetes mellitus, preeclampsia was not associated with weight gain during pregnancy after diagnosis of gestational diabetes mellitus.

Regarding the risk of gestational hypertension, the systematic review and meta-analysis of Zhu et al. [12] including pregnant women from China with gestational diabetes mellitus, concluded similar results to ours, whereas EGWG was associated with a higher risk of the aforementioned complication.

Regarding the increased risk of delivery by cesarean section, the systematic review and meta-analysis of Zhu et al. [12] including pregnant women from China with gestational diabetes mellitus and meta-analysis of Rogozinska [16] which analyzed individual participant data of healthy women with a singleton pregnancy from 36 randomized trials (16 countries) concluded similar results to the present review. In two other systematic reviews [13,14], one including pregnant women with gestational diabetes mellitus and one regarding twin pregnancies, the association between the risk of delivery by cesarean section and EGWG was found to be statistically insignificant.

The risk of prenatal depressive symptoms was found to be increased with EGWG in the present review, but systematic reviews and meta-analyses regarding perinatal complications that were identified did not examine this specific perinatal complication.

Our results about the risk of macrosomia-high birthweight of the neonate and EGWG were found to be conflicting, whereas the risk of macrosomia was increased in the overall population of all included studies but decreased in one included study for pregnant women who were underweight before pregnancy. Thus, our finding of macrosomia’s decreased risk with EGWG should be interpreted with skepticism, as it comes from a single study. The increased risk of macrosomia in our review is aligned with the results of Perumal et al.’s meta-analysis [17] including data from 24 low- and middle-income countries. Nevertheless, Asefa’s systematic review and meta-analysis including data from sub-Saharan Africa concluded with statistically insignificant results regarding preterm birth and EGWG, despite that this finding comes from only two included studies [15].

Our results about the EGWG and increased risk of a large-for-gestational-age neonate are in accordance with the results of four other systematic reviews and meta-analyses: the review and meta-analysis of He et al. [14] regarding pregnant women with gestational diabetes mellitus, meta-analysis of Perumal et al. [17] including data from 24 low- and middle-income countries, systematic review and meta-analysis of Zhu et al. [12] including pregnant women from China with gestational diabetes mellitus and analysis of Rogozinska [16] which analyzed individual participant data of healthy women with a singleton pregnancy from 36 randomized trials (16 countries). These findings could be explained by the positive and causal association between pregnancy weight gain and fetal size that has been found using a sibling comparison design [24].

The reduced risk of a small-for-gestational-age neonate that was observed in this systematic review is aligned with two other systematic reviews–meta-analyses: the systematic review and meta-analysis of Zhu et al. [12] including pregnant women from China with gestational diabetes mellitus and meta-analysis of Rogozinska [16] which included individual participant data (IPD) of healthy women with a singleton pregnancy from 36 randomized trials (16 countries). Contrary to that, two other reviews (the systematic review and meta-analysis of Lipworth et al. [13] regarding twin pregnancies and review and meta-analysis of He et al. [14] regarding pregnant women with gestational diabetes mellitus) found a statistically insignificant association.

The risk of the neonate’s low birthweight was found to be decreased with EGWG in the present review, but systematic reviews and meta-analyses regarding perinatal complications that were identified did not examine this specific perinatal complication.

We found an increased risk of neonatal hypoglycemia requiring treatment with EGWG, but contrary to our results, the review and meta-analysis of He et al. [14] regarding pregnant women with gestational diabetes mellitus concluded statistically insignificant results.

### 4.1. Strengths of the Present Review

Considering that a proper clinical trial could not be conducted due to ethical limitations (excessive maternal weight gain during pregnancy is likely to cause negative consequences to the pregnant woman’s and neonate’s health), the highest and at the same time the most appropriate study design for this type of clinical question is a cohort study. Thus, only grade III studies (prospective cohort studies), according to the hierarchical system “Levels of Evidence” [25], were included. To our knowledge, the present systematic review provides the most recent and strong evidence regarding the correlation between EGWG and adverse perinatal outcomes.

### 4.2. Limitations of the Present Review

Several factors related to the characteristics of the included studies may influence the results of this review. One of the most important is that three out of five included studies are from countries in East Asia (China, Taiwan, Korea). That means the results of these three studies are likely to be influenced by the characteristics of this particular racial/ethnic group regarding the increased risk of perinatal complications such as preterm birth and small-for-gestational-age neonates [26]. In addition, no studies from countries in Europe and Africa were identified. Some of the included studies were based on self-reporting regarding the evaluation of the exposure and not on direct measurement by medical staff. Specifically, this included recording the last weight that pregnant women remembered to have before their pregnancy and not its direct measurement during the first prenatal visit. A final factor that probably plays a crucial role is that the included studies omitted the collection of data about other adverse perinatal outcomes such as postpartum hemorrhage, premature rupture of fetal membranes and complications that concern exclusively the neonate such as the need for mechanical ventilation, jaundice requiring phototherapy, etc. However, the aforementioned perinatal complications were examined by retrospective cohort studies that were excluded due to their design [27,28,29,30,31,32].

Additionally, the results of this review are likely to be affected by various limitations related to the methodology followed, except the characteristics of the included studies. One limitation is that the included studies are published—as full text—only in the English language. Thus, studies published in any other language were excluded even though they may contain important information relevant to the topic. However, only a small number of relevant reports (two) that met the inclusion criteria were published in other languages (Chinese, French). Moreover, since the review was limited to the last five years, the small number of included studies is a limitation. However, the fact that the included studies were of high quality is a strength of the present review.

### 4.3. Clinical Practice and Future Directions

The results of the present review suggest that, in daily clinical practice, particular emphasis should be given to adhering to guidelines for appropriate weight gain in women who already have modifiable risk factors such as overweight and obesity before pregnancy. According to a recent meta-analysis using data from 196,670 participants within 25 cohort studies from Europe and North America [33], optimal gestational weight gain ranges were 14.0 kg to less than 16.0 kg for women categorized as underweight; 10.0 kg to less than 18.0 kg for normal weight; 2.0 kg to less than 16.0 kg for overweight; 2.0 kg to less than 6.0 kg for obesity grade 1; weight loss or gain of 0 kg to less than 4.0 kg for obesity grade 2; and weight gain of 0 kg to less than 6.0 kg for obesity grade 3. Institute of Medicine 2009 guidelines [3] recommend a less strict range for gestational weight gain; for a singleton pregnancy, they recommend underweight women to gain between 12.5 and 18 kg, normal-weight women between 11.5 and 16 kg, overweight between 6.8 and 11.3 and obese between 5 and 9 kg.

According to this review and as the absence of recent and well-designed prospective cohort studies is evident in Europe and Africa, large studies are necessary in these racial/ethnic groups. Finally, as the results regarding the risk of preterm birth and macrosomia-high birth weight of the neonate are conflicting, it may be useful to further investigate these two adverse outcomes in various subgroups of the pregnant population (underweight, overweight and obese before pregnancy).

## 5. Conclusions

Excessive gestational weight gain, as defined by official medical organizations for each country/region and based on a woman’s body mass index status before each pregnancy, appears to have a detrimental effect on most perinatal complications. It increases the risk of various adverse perinatal outcomes for the mother’s and neonate’s health, according to this systematic review. The risk of hypertensive disorders of pregnancy is the most serious burden regarding the pregnant woman’s health, especially for those who already have risk factors such as obesity. However, in very few complications (low birth weight of the neonate, small-for-gestational-age neonate), this excessive weight gain was found to exert a clearly protective effect. The highest protective effect is shown regarding small-for-gestational-age neonates. This systematic review, to our knowledge, is the most recent, up-to-date and thorough review on this topic.

## Figures and Tables

**Table 1:** Characteristics of the included studies.

Study	Study Type	Geographical Area of the Study	Sample	Perinatal Outcomes Assessed by the Study	Weight Gain in Pregnancy in Kilograms	Gestational Age at Delivery in Weeks	Mode of Delivery	Neonatal Birth Weight in Grams	Apgar Score
Bouvier et al., 2019 [18]	Prospective cohort study	Canada, North America	7866 pregnant women > 18 years old	1. Gestational diabetes mellitus 2. Hypertensive disorders of pregnancy (gestational hypertension and preeclampsia) 3. Mode of delivery (cesarean section or not) 4. Neonatal macrosomia 5. Large-for-gestational-age neonate 6. Small-for-gestational-age neonate 7. Neonatal hypoglycemia requiring treatment	Median (min, max, interquartile range) First trimester Profile A (85.6% were of normal weight): 2 (−9.5, 21, 1–3.7) Profile B (50.9% were overweight): 2.7 (−9, 20.6, 1–4.9) Profile C (89.3% were obese): 1.7 (−10.8, 28.6, 0–4) Second trimester Profile A (85.6% were of normal weight): 8.8 (−5, 28, 6.8–11) Profile B (50.9% were overweight): 9.5 (−11, 28, 7–12.8) Profile C (89.3% were obese): 7 (−9.6, 38.7, 3.5–11) Third trimester Profile A (85.6% were of normal weight): 14 (−0.5, 40.3, 11.6–17) Profile B (50.9% were overweight): 15.9 (−11.1, 44, 12–19.5) Profile C (89.3% were obese): 13 (−9.6, 45.7, 7.7–17.7)	Gestational weight gain above IOM recommendations in the second trimester of pregnancy: mean (standard deviation)—39.4 (1.5) Gestational weight gain above IOM recommendations in the third trimester of pregnancy: mean (standard deviation)—39.5 (1.3)	Not stated	Not stated	Not stated
Chen et al., 2020 [19]	Prospective cohort study	Taiwan, East Asia	19,052 pregnant women from 369 cities and regions	1. Gestational diabetes mellitus 2. Gestational hypertension 3. Preeclampsia 4. Delivery by cesarean section 5. Preterm birth (gestational age < 37 weeks) 6. Low birth weight of the neonate (<2500 g) 7. Macrosomia-high birth weight of the neonate (>4000 g)	Not stated	Not stated	Not stated	Not stated	Not stated
Choi et al., 2022 [20]	Prospective cohort study	Korea, East Asia	3454 pregnant women with singleton pregnancies	1. Hypertensive disorders of pregnancy (gestational hypertension, preeclampsia, eclampsia, superimposed preeclampsia) 2. Gestational diabetes mellitus 3. Perinatal depressive symptoms 4. Prenatal depressive symptoms 5. Postpartum depressive symptoms 6. Delivery by cesarean section 7. Complications during normal labor 8. Premature birth 9. Low birth weight of the neonate (<2500 g) 10. High birth weight of the neonate (>4000 g) 11. Admission to the Neonatal Intensive Care Unit	Mean ± standard deviation—13.2 ± 4.7	Mean ± standard deviation—39.3 ± 1.4	Cesarean delivery number (percentage %)— 1374 (39.8)	Not stated	Not stated
Mosquera et al., 2022 [21]	Prospective cohort study	Brazil, South America	1305 mother–child pairs	1. Maternal hemoglobin levels 2. Gestational anemia 3. Delivery by cesarean section 4. Premature birth 5. Neonatal macrosomia 6. Low birth weight of the neonate 7. Large-for-gestational-age neonate 8. Small-for-gestational-age neonate	Not stated	Number (percentage %) <37 weeks at delivery 91 (7.0) ≥37–41 weeks at delivery 1180 (90.4) ≥42 weeks at delivery 34 (2.6)	Not stated	Not stated	Not stated
Zheng et al., 2021 [22]	Prospective cohort study	China, East Asia	3126 pregnant women with gestational diabetes mellitus	1. Gestational hypertension and preeclampsia 2. Neonatal macrosomia 3. Low birth weight of the neonate 4. Large-for-gestational-age neonate 5. Small-for-gestational-age neonate 6. Premature birth 7. Delivery by cesarean section	Mean ± standard deviation—13.10 ± 5.26	Mean ± standard deviation—38.58 ± 1.41	Cesarean section number (percentage%)— 1102 (35.25)	Mean ± standard deviation—3412 ± 483	Not stated

**Table 2:** Assessment of methodological quality of the included studies.

Assessment Parameters of the Newcastle–Ottawa Scale		(Bouvier, Forest et al., 2019) [18]	(Chen, Chen et al., 2020) [19]	(Choi, Lim et al., 2022) [20]	(Zheng, Huang et al., 2021) [22]	(Mosquera, Malta et al., 2022) [21]
SELECTION (maximum: 4 *)	Representativeness of the intervention cohort ^a^	*	*	*	*	*
	Selection of the non-intervention cohort ^b^	*	*	*	*	*
	Ascertainment of intervention ^c^	*	-	*	*	*
	Demonstration that outcome of interest was not present at start of study ^d^	*	*	-	*	-
COMPARABILITY (maximum: 2 *)	Comparability of cohorts on the basis of the design or analysis ^e^	**	**	**	**	**
OUTCOME (maximum: 3 *)	Assessment of outcome ^f^	*	-	*	*	*
	Follow-up long enough for outcomes to occur ^g^	*	*	*	*	*
	Adequacy of follow-up of cohorts ^h^	*	*	*	*	*
Methodological quality of the study		9*/High	7*/High	8*/High	9*/High	8*/High

a. * Representative of the excessive weight gain during pregnancy and perinatal outcomes, - somewhat representative or selected groups, n/a no description of the derivation of the cohort. b. * Drawn from the same source as the cohort, - drawn from a different source, n/a no description of the derivation of the non-exposed cohort. c. * Secure record, - interviews or self-reports, n/a no description. d. * Report that the outcome was not present at the start of the study, - no report that the outcome was not present at the start of the study. e. ** Study controls for the most important factor, * study controls for any additional factor, n/a not carried out or not reported. f. * Independent blind assessment, - missing records or self-report, n/a no description. g. * Follow-up was long enough for outcomes to occur, - follow-up was not long enough for outcomes to occur. h. * all the samples were followed up, - subjects lost at follow-up, n/a not mentioned in the study.

**Table 3:** Effect of excessive gestational weight gain on increase or decrease in risk for each type of adverse perinatal outcome.

Type of Adverse Perinatal Outcome	Risk of Adverse Perinatal Outcome	Summary of Studies Supporting This Particular Effect (↑ or ↓ Risk of Each Complication)
Gestational diabetes mellitus	↑ by 2.76 times in pregnant women who were overweight before pregnancy [19] to 3.53 times in pregnant women who were obese before pregnancy [19]	1 [19]
Preterm birth	↓ by 20% [19]	1 [19]
	↑ by 49% in women who were overweight before pregnancy [19] to 95% in women who were obese before pregnancy [19]	1 [19]
Any hypertensive disorder of pregnancy	↑ by 80% in pregnant women with excessive weight gain in the 2nd trimester of pregnancy [18] up to 3253 times [20]	4 [18,19,20,22]
Gestational hypertension	↑ by 2.51 times [19] to 6.2 times in pregnant women who were obese before pregnancy [19]	1 [19]
Preeclampsia	↑ by 3.17 times [19] to 3.3 times in pregnant women who were obese before pregnancy [19]	1 [19]
Delivery by cesarean section	↑ by 21% in pregnant women with excessive weight gain in the 2nd trimester of pregnancy [18] up to 3.52 times in women with gestational diabetes mellitus [22]	5 [18,19,20,21,22]
Prenatal depressive symptoms	↑ by 30% [20]	1 [20]
Macrosomia-high birthweight of the neonate	↓ by 48% in pregnant women who were underweight before pregnancy [19]	1 [19]
	↑ by 66.6% [20] up to 2.66 times [19]	5 [18,19,20,21,22]
Large-for-gestational-age neonate	↑ by 57% in pregnant women with excessive weight gain in the 2nd trimester of pregnancy [18] up to 2.26 times in pregnant women with excessive weight gain in the 3rd trimester of pregnancy [18]	3 [18,21,22]
Small-for-gestational-age neonate	↓ by 39% in pregnant women with excessive weight gain in the 2nd trimester of pregnancy [18] to 62% [21]	3 [18,21,22]
Low birthweight of the neonate	↓ by 26% [19] to 56% [21]	3 [19,20,21]
Neonatal hypoglycemia requiring treatment	↑ by 89% in pregnant women with excessive weight gain in the 3rd trimester of pregnancy [18]	1 [18]

## Data Availability

Data are contained within the article or Supplementary Material.

## Supplementary Materials

The following supporting information can be downloaded at https://www.mdpi.com/article/10.3390/jcm14041197/s1, Table S1: Detailed search strategy used for each electronic database.
