# Supplementary material for: The Impact of Excessive Gestational Weight Gain on Adverse Perinatal Outcomes: A Systematic Review
Source: J Clin Med. 2025 Feb 12;14(4):1197. doi: 10.3390/jcm14041197 (PMC11856671; doi:10.3390/jcm14041197)
Supplement: Supplementary file 1 [file jcm-14-01197-s001.zip › jcm-3394623-supplementary.pdf]

Supplementary Table S1. Detailed search strategy used for each electronic database

Medline/PubMed
The full search strategy for the Medline/PubMed database used was:<br>1. (excessive weight gain[Title]) OR (weight gain[Title]) OR (BMI change[Title]) OR (body mass index change[Title]) AND (perinatal adverse outcomes[Title/Abstract]) AND (y_5[Filter])<br>2. (excessive weight gain[Title]) OR (weight gain[Title]) OR (BMI change[Title]) OR (body mass index change[Title]) AND (adverse perinatal outcomes[Title/Abstract]) AND (y_5[Filter])<br>3. (excessive weight gain[Title]) OR (weight gain[Title]) OR (BMI change[Title]) OR (body mass index change[Title]) AND (perinatal complications [Title/Abstract]) AND (y_5[Filter])
Scopus
For the Scopus database the full search strategy was:<br>(TITLE (weight AND gain) OR TITLE (excessive AND weight AND gain) OR TITLE (bmi AND change) OR TITLE (body AND mass AND index AND change) OR TITLE (weight AND retention) OR TITLE (excessive AND weight AND retention) AND TITLE (perinatal AND adverse AND outcomes) OR TITLE (adverse AND perinatal AND outcomes) OR TITLE (perinatal AND complications)) AND PUBYEAR > 2013 AND PUBYEAR < 2025
CADTH Grey Matters database
For the CADTH Grey Matters database, the phrases “perinatal outcomes” OR “pregnancy outcomes” OR “birth outcomes” OR “neonatal outcomes” AND “weight gain” OR “weight” OR “BMI” OR “body mass index” were used in the “Title” field
National Archive of Doctoral Dissertations (NAD)
For the National Archive of Doctoral Dissertations (NAD) database, the combination of the phrases “weight gain” or “weight” or “body mass index” or “BMI” AND “perinatal complications” or “pregnancy complications” or “birth complications” AND in the “Abstract” field "date" the criterion "after 2018"
